# The effects of acute wild blueberry supplementation on the cognition of 7–10-year-old schoolchildren

**DOI:** 10.1007/s00394-018-1843-6

**Published:** 2018-10-16

**Authors:** Katie L. Barfoot, Gabrielle May, Daniel J. Lamport, Jessie Ricketts, Patricia M. Riddell, Claire M. Williams

**Affiliations:** 1 School of Psychology and Clinical Language Sciences, University of Reading, Earley Gate, Whiteknights Road, Reading, RG6 6AL UK; 2 School of Psychology, Royal Holloway, University of London, London, UK

**Keywords:** Cognition, Memory, Attention, Reading, Flavonoid, Blueberries, Children

## Abstract

**Purpose:**

Previous evidence suggests consumption of flavonoids, a sub-class of polyphenols, is associated with improved cognitive function across the lifespan. In particular, acute intervention of a flavonoid-rich wild blueberry (WBB) drink has been shown to boost executive function (EF), short-term memory and mood 2–6 h post-consumption in 7–10-year-old children. However, confirmation of the aspects of EF and memory susceptible to WBB ingestion is required, particularly during childhood, a critical period of neurological development. In addition, the child literature on berry flavonoid supplementation and cognition highlights the potential for such interventions to elicit positive benefits to real-world educational scenarios, such as reading, a complex ability which relies upon aspects of cognition already known to improve following WBB.

**Methods:**

Here we examined which aspects of EF and memory are susceptible to acute WBB, as well as investigating whether acute WBB could further benefit reading ability. Fifty-four healthy children, aged 7–10 years, consumed a 200 ml WBB drink (253 mg anthocyanins) or a matched placebo according to a randomised, single-blind, parallel-groups design. Verbal memory (Auditory Verbal Learning Task; AVLT), EF (Modified Attention Network Task; MANT), and reading efficiency (Test of Word Reading Efficiency-2; TOWRE-2) were assessed at baseline and 2 h post-consumption.

**Results:**

For the MANT, significantly quicker RTs were observed for WBB participants when compared to placebo participants on 120 ms trials, without cost to accuracy. Furthermore, WBB participants showed enhanced verbal memory performance on the AVLT, recalling more words than placebo participants on short delay and memory acquisition measures post-consumption. Despite these significant improvements in cognitive performance, no significant effects were observed for reading measures.

**Conclusion:**

Consumption of WBB was found to significantly improve memory and attentional aspects of EF. This indicates that a flavonoid-rich blueberry product, equivalent to 240 g or 1½ cups of fresh blueberries can provide acute cognitive benefits in children. These findings support accumulating evidence that flavonoid-rich products are beneficial for healthy brain function, particularly during critical developmental periods. However, the lack of findings relating to reading ability suggested acute WBB may not be sufficient to elicit benefits to reading. Chronic supplementation and other more sensitive reading measures should be considered for examining the effects of WBB on such a complex skill in the future.

## Introduction

The health benefits of berry fruits are well established, with positive outcomes from consumption associated with cardiovascular health [1], diabetes [2] and cancer prevention [3]. These benefits are thought to be mediated by the naturally high concentrations of anthocyanins, a sub-class of flavonoids from plant-based polyphenols, found in such fruits [4, 5]. In addition, berry fruit consumption has been associated with cognitive benefits. Rodent models have demonstrated significant improvements in working and short-term memory [6, 7], and reversal of age-related cognitive decline in aged animals [8, 9]. Positive cognitive effects have also been observed throughout the human lifespan [10, 11]. The neuroprotective findings from rodent models are mirrored in older adults with a reduction of cognitive impairments and delayed onset of ageing disorders, such as Alzheimer’s disease [12–16]. Additionally, acute and chronic berry flavonoid interventions enhance cognition in healthy older and young adults [17–20] which builds upon the wider flavonoid literature for these populations [21–25]. Recently, berry flavonoid interventions have shown significant improvements in mood for both adolescents and school-aged children [26].

It is of interest to explore whether such cognitive benefits extend to child populations. For example, Calderón-Garcidueñas et al. [27] first showed that children (mean age 10 years) supplemented daily with 680 mg cocoa-flavanols over 9–24 days exhibited significant improvements in short-term memory assessed by letter and object span tests. In a further acute study, Whyte and Williams [28] observed significant improvements in 8–10-year-olds’ word recall performance for both short and long delays at 2 h post-consumption of a 200 g fresh blueberry drink (143 mg anthocyanins). In a similar study, the cognitive performance of 7–10-year-olds was examined at baseline, 1.15 h, 3 h and 6 h post-consumption of two wild blueberry (WBB) interventions: a 30 g WBB drink (253 mg anthocyanins) and a 15 g WBB drink (127 mg anthocyanins), compared to a sugar-matched placebo drink [29]. In line with previous research, significant WBB-related improvements were observed for word recognition at every time point and delayed word recall at 1.15 h only. Additionally, significant improvements in accuracy on a cognitively-demanding executive function task were observed at 3 h post-consumption of the 30-g WBB drink. These findings highlight the potential for WBB interventions to enhance word recall and recognition performance, as well as improving executive function up to 6 h post-consumption in 7–10-year-olds. At this age, children experience a spurt in frontal lobe growth thought to coincide with enhanced executive functions and the progression of cognitive abilities [30, 31], a phase of cognitive development where WBB supplementation may be particularly beneficial. Indeed, Whyte, Schafer and Williams [32] observed significantly faster reaction times on executive function trials requiring greater cognitive demand (incongruent, high load) at 3 h post-WBB consumption compared to placebo in 7–10-year-olds, thus demonstrating WBB benefits children’s executive function when performing complex and demanding cognitive tasks.

In the present study, we aim to replicate the findings from Whyte, Schafer and Williams [29, 32] on memory and executive function following an acute high-flavonoid WBB intervention in 7–10-year-olds. Additionally, given the boosts in cognitive performance seen after flavonoid intervention, Whyte and colleagues further suggested that such positive findings from acute WBB consumption on cognition has the potential to benefit a real-world educational scenario. We aimed to extend these cognitive findings in a learning environment by further examining whether acute WBB interventions can benefit reading ability in this population. Reading is a complex process requiring multiple aspects of cognition (e.g. memory, attention, executive functions) which are required for skills such as word learning (decoding), recognition, vocabulary knowledge and reading comprehension. Indeed, previous cognitive findings [28, 29] demonstrate improvements to word recall and recognition suggesting the potential for berry flavonoids to benefit children’s visual recall ability of words stored in their internal lexicon; an ability known as sight word reading [33], and to executive functions thought to play a vital role in word learning and reading comprehension [34]. To date, the role of berry flavonoids on reading ability has not yet been examined; however, research does demonstrate a number of links between better executive function and academic performance in general. For example, greater working memory and inhibition are found to be predictive of greater maths and reading ability in 9–12-year-old school children [35, 36], as well as a positive association between greater processing speed and greater performance for maths, reading ability and language skills in 6–19-year-olds [37, 38]. Therefore, as previous berry flavonoid interventions in children have been shown to positively benefit executive function, in addition to the above literature demonstrating improvements to reading and other academic skills in line with improvements in executive functions, we hypothesise that improvements in children’s reading ability following acute WBB intervention will be observed.

## Methods

### Participants

An *a priori* power analysis (using G Power 3.1.), based on previous significant findings from our laboratory [29] using an effect size of 0.22, was conducted using F test, repeated measures, within–between interaction parameters. The analysis concluded that 50 participants (*n* = 25 per condition) would be required to achieve a power of 0.85 when completing two repetitions (baseline, post-consumption) [*F*(1,48) = 4.04]. A total of 54 healthy participants (25 male: 29 female) aged 7–10 years old (*M* = 8.24, SD = 0.97) of any ethnicity were recruited from two primary schools. Written consent was obtained from parents/legal guardians prior to the child’s participation. Parents/legal guardians confirmed that the children spoke English as a first language, had not been diagnosed with attention-deficit/hyperactivity disorder (ADHD) or a reading impairment, and had no known fruit or fruit juice intolerances. All participants followed a low-flavonoid diet 24 h prior to the main test day; for further information see “Procedure”.

### Treatments

An acute, single-blind, randomised, parallel-groups design was applied, with participants randomly allocated to receive either 30 g freeze-dried wild blueberry (WBB) drink or a sugar-matched placebo. The 30 g WBB treatment was equivalent to 240 g fresh blueberries or 1½ cups of fresh blueberries and contained 253 mg anthocyanins. The placebo contained fructose (8.9 g), glucose (7.99 g) and vitamin C (4 ml) to match the concentrations found in the 30 g WBB treatment. To aid consumption and palatability, 170 ml of cold tap water and 30 ml of a low-flavonoid fruit squash (Rocks brand, UK) were added to both treatments, producing a 200 ml drink. Treatments were prepared immediately before being administered to participants in an opaque drinking flask and consumed through an opaque straw to ensure the participants remained blind to the treatment. Participants were given 5 min to consume the drink, to which all complied.

### Cognitive tests

1. *Reys Auditory Verbal Learning Task (AVLT)*—participants heard an auditory recording of 15 nouns (list A), read at 1 per second. Each presentation was followed by a free recall of this list (recalls A1–A5). A new list of 15 nouns (list B) was introduced as an interference list on trial 6 and was recalled once only (recall B). Participants then recalled list A after a short delay (2 min) and a long delay (15 min) (free recalls A6 and A7). After recall A7, participants were visually presented with 50 nouns containing: words from lists A and B, and 20 additional nouns. They were asked to circle words from list A only. Word list versions were counterbalanced across test sessions.

For each test session, a series of outcome measures were calculated according to Lezak [39] and previous research [28]: immediate word span (recall A1); words learnt (recall A5–recall A1); final acquisition (recall A5); proactive interference (recall A1–recall B); retroactive interference (recall A5–recall A6); word recognition (the number of words correctly circled); total acquisition (sum A1 through A5); total recall A (sum A1 through A7); total recall A&B; and total delayed recall (A6 + A7).

2. *Modified Attention Network Task (MANT)*—this task combines a cue-target and flanker task to measure vigilance, selective attention and response interference under conditions of differing cognitive demand. Here, stimuli load, duration, orientation and cueing were manipulated to change the cognitive demand of the task. In accordance with Whyte et al. [29], a practice block of 35 trials was initially completed where target duration decreased from 1000 to 120 ms over 16 trials to allow familiarisation with the speed of response required. Subsequently, participants completed four target blocks of the MANT each consisting of 80 trials.

For the main target blocks, a fixation slide was first shown with a cross displayed centrally. This was followed by either no cue, a central cue, a double cue or a spatial cue for 120 ms with cue location (above or below the central fixation cross) randomised between trials. The target stimulus, a single arrow pointing “<” or “>”, was then displayed either above or below the fixation cross for either 120 ms (two target blocks) or 500 ms (two target blocks); stimulus location was not always consistent with prior cue location. The target stimulus appeared individually or surrounded by pairs of flanker arrows, and could be congruent (i.e. <<<<< or >>>>>) or incongruent (i.e. <<><< or >><>>) with the surrounding arrows. Individual arrows exhibited low load whilst congruent or incongruent trials either exhibited medium load (one row of five arrows) or high load (two rows of five arrows). The stimulus position, congruence and load were randomised between trials, displayed in equal probability. Additionally, one 120 ms target block and one 500 ms target block were presented with playground noise, increasing cognitive demand of these trials. Participants were instructed to press the left or right arrow key on the keyboard according to the direction of the target stimulus arrow for each trial.

The outcome measures for the MANT were accuracy (number of correct hits) and reaction time for correct targets (speed of response to target; with reaction times < 200 ms removed).

3. *Test of Word Reading Efficiency (TOWRE-2)—*the TOWRE-2 consists of two subtests: sight word efficiency (SWE) and phonemic decoding efficiency (PDE) [40]. The SWE subtest measures word reading efficiency, whilst the PDE subtest measures non-word reading efficiency. For both, participants are visually presented with 108 words or 63 non-words, increasing in difficulty from single-syllabic to multisyllabic, and are asked to read as many items as possible in 45 s. Each subtest has four parallel forms which were randomised between participants and sessions.

The outcome measures for the TOWRE-2 were number of words (SWE) and non-words (PDE) pronounced correctly in 45 s.

Finally, subjective mood data using the child version of the Positive and Negative Affect Scale (PANAS-C) were collected and is presented elsewhere [26].

### Procedure

Practice session: participants took part in a practice session 1–2 days prior to the test day. Here, participants completed a practice of the AVLT and MANT. At the same time, participants completed the demographic measures: a children’s version of Ravens Coloured Progressive Matrices (RCPM) as a measure of fluid intelligence, a Continuous Performance Task (CPT) as a measure of sustained attention, the York Assessment of Reading for Comprehension (YARC) as a measure of general reading ability, and the Single Word Reading Task (SWRT) as a measure of word reading accuracy. Both raw score and standard score means, and standard deviations for these are shown in Table 1. Participants also completed a practice of the main cognitive test battery. To avoid the YARC and SWRT interfering with the AVLT practice, these two demographic measures were completed last. Finally, all participants were reminded to comply with the 24-h low-flavonoid diet. An information sheet detailing high-flavonoid foods to avoid (berry fruits, general fruit and vegetables, chocolate, to name a few) and low-flavonoid alternatives was given to parents/legal guardians to ensure compliance. Compliance and adherence to the restrictions were orally checked with children, parents and canteen staff on the test days.

**Table 1:** Demographic and screening data for placebo and WBB participants

	Placebo (*n* = 25)		WBB (*n* = 29)		
	Mean	SD	Mean	SD	*P* statistic
Age	8.23	1.05	8.24	0.88	0.97
Gender (M:F)^a^	13:12	–	12:17	–	–
School year (3:4:5)	8:7:8	–	8:7:10	–	–
CPT	(*n* = 22)		(*n* = 28)		
Omissions (%)	10.74	7.14	11.21	16.03	0.90
Commissions (%)	88.48	8.21	89.17	15.62	0.85
RCPM	(*n* = 22) 26.55	6.10	(*n* = 27) 26.78	4.40	0.88
YARC					
Reading accuracy	(*n* = 24)		(*n* = 27)		
Raw scores	14.5	7.93	17.48	9.14	0.32
Standard scores	103.37	16.96	107.74	11.27	0.28
Reading rate					
Raw scores (ms)	286.36	92.69	293.44	90.07	0.45
Standard scores	107.09	15.72	109.81	11.73	0.50
Reading comprehension					
Raw scores	11.36	2.88	11.33	2.13	0.96
Standard scores	111.12	17.81	115.51	13.82	0.32
SWRT					
Raw scores	40.25	11.82	45.18	8.89	0.09
Standard scores	103.91	19.56	111.4	13.73	0.07

No significant differences between treatment groups were seen for the demographic measures or performance of the CPT, RCPM, YARC or SWRT. YARC standard scores: mean = 100, SD = 15

^a^No significant differences were observed between the number of males and females in each treatment group (males, *χ*^2^ = 0.15, *p* = 0.70; females, *χ*^2^ = 0.86, *p* = 0.35)

### Test day

Testing took place during the afternoon, after lunch, individually in a quiet space at their schools. Participants consumed either a low-flavonoid packed lunch provided by parents or a low-flavonoid cooked lunch provided by the school canteen on the day of testing. The specific content of the lunch was not standardised or recorded. Lunch was consumed in the 1.15-h period prior to baseline testing. The initial baseline session commenced at 1200 h, 1245 h or 1330 h immediately before consuming the intervention or placebo drink. After consumption, all children returned to class for a 2-h period during which time they were only allowed to consume water and abstained from exercise. Cognitive performance was assessed at 2 h post-consumption for consistency with Whyte et al. [29]; this is where polyphenol metabolites from WBB are known to peak [41] leading to their optimum absorption and metabolism within the body. Therefore, the second test session, post-drink consumption, took place at 1440 h, 1525 h or 1610 h (dependent on time of consumption of the intervention). Here, participants completed a different version of the same test battery to assess any changes in performance. During each test session, participants completed the tasks in the following order: AVLT recalls 1–6, MANT, AVLT recall 7, word recognition, and TOWRE-2. Test versions of equivalent difficulty were counterbalanced across visits and conditions. Each session lasted approximately 40 min.

### Statistical analysis

Data were analysed using SPSS (Version 21.0). Baseline differences in non-verbal ability (RCPM), attention (CPT commissions, CPT omissions), general reading ability (YARC reading accuracy, reading rate, reading comprehension), and word reading accuracy (SWRT) were examined using t-tests with drink (placebo, WBB) as the independent variable. YARC standard scores were calculated by adding together the raw scores from two passages to give an average ability, and comparing this to participant’s age using the conversion tables in the YARC manual to produce standard scores, used for analysis. Two chi-square tests were performed to assess sex differences across the treatment groups. All other data were analysed using linear mixed-effects models (LMMs) with baseline performance included as a fixed factor. This technique was used to account for the dependent nature of data points in the repeated measures design, and to avoid exclusion of missing data. LMMs were performed using an unstructured covariance matrix, as covariances were deemed unpredictable, with no fixed underlying structure. Separate LMMs were performed for each dependent variable (DV).

Drink (placebo, WBB) was included as fixed factors in all LMMs to compare the effects of treatment. Participants were also included as a random factor to control for dependence of data within-subjects. For the MANT, congruency (congruent, incongruent), load (high load, medium load) and target time (120 ms, 500 ms) were also included as fixed factors in the model to detect changes in relation to cognitive load. These factors were also individually combined with drink (placebo, WBB) to assess any interactions between performance and treatment group (drink × congruence, drink × load, drink × target time). Pairwise comparisons were used to explore significant effects, and were corrected for type 1 errors using Bonferroni adjustment.

## Results

There were no significant differences at baseline between groups for RCPM (*t*(47) = − 0.16, *p* = 0.88), CPT commissions (t(46) = − 0.19, *p* = 0.85), CPT omissions (*t*(46) = − 0.13, *p* = 0.90) or age (*t*(52) = − 0.034, *p* = 0.97; see Table 1). There were also no significant differences between groups at baseline for MANT accuracy (*t*(52) = − 0.156, *p* = 0.88), MANT RT (*t*(52) = 0.87, *p* = 0.39) or for any AVLT list recalls.

### Modified attention network task (MANT)

#### Reaction time (RT; ms)

Target time (*F*(1,396.13) = 0.56, *p* = 0.45) and drink (*F*(1,45.57) = 1.63, *p* = 0.21) did not independently predict RT performance; however, there was a significant fixed effect interaction whereby drink x target time predicted post-consumption RT (*F*(367.82) = 5.63, *p* = 0.018).

**Fig. 1:**
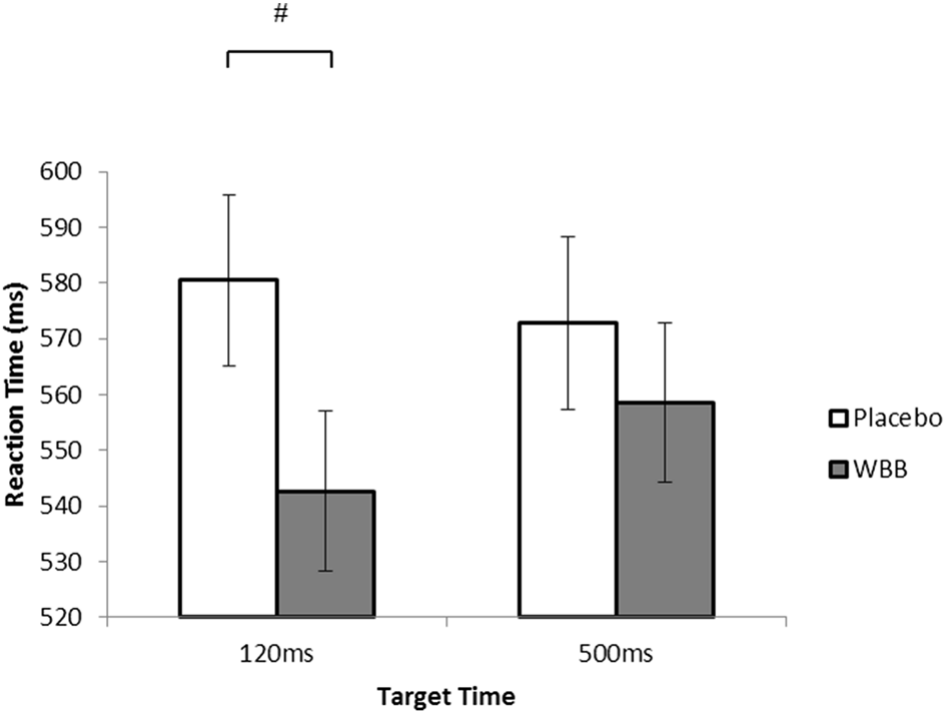
Mean post-consumption MANT RT scores of placebo and WBB treatment drinks on 120 ms and 500 ms trials. WBB participants perform faster on 120 ms trials than placebo participants

Pairwise comparisons exploring differences between treatment for each target time variable revealed a trend whereby WBB participants (*M* = 542.59, SE = 14.39) performed quicker than placebo participants (*M* = 580.45, SE = 15.42) on 120 ms trials (*p* = 0.078; Fig. 1). This suggests WBB consumption increased mental alertness for 120-ms trials, without changing on 500 ms trials.

As expected, congruency was a significant predictor of performance, such that participants were significantly quicker on congruent trials (*M* = 539.32, SE = 10.58) than incongruent trials (*M* = 587.82, SE = 10.58), regardless of treatment (F (1,398.34) = 77.34, *p* < 0.01). Similarly, load was a significant predictor of performance, such that RT on medium-load trials (*M* = 558.48, SE = 10.51) was significantly quicker than on high-load trials (*M* = 568.66, SE = 10.51), regardless of treatment (*F*(1,369.72) = 4.19, *p* = 0.04).

#### Accuracy (%)

Drink was not a significant predictor of accuracy performance (*F*(1,48.52) = 0.17, *p* = 0.68). There were no significant fixed effect interactions. As expected [32], congruency was a significant predictor of performance, such that participants were more accurate on congruent trials (*M* = 0.85, SE = 0.017) than incongruent trials (*M* = 0.79, SE = 0.017), regardless of treatment (*F*(1,404.22) = 26.70, *p* < 0.01). Similarly, load was a trending predictor of performance, such that accuracy on medium-load trials (*M* = 0.83, SE = 0.016) was better than high-load trials (*M* = 0.81, SE = 0.016), regardless of treatment (*F*(1,371.98) = 3.55, *p* = 0.06). Target time was also a significant predictor of performance, such that accuracy on the more cognitively demanding 500 ms trials (*M* = 0.84, SE = 0.017) was better than the less demanding 120 ms trials (*M* = 0.80, SE = 0.017), regardless of treatment (*F*(1,377.92) = 15.49, *p* < 0.01).

### Auditory Verbal Learning Task (AVLT)

#### AVLT drink effects

**Fig. 2:**
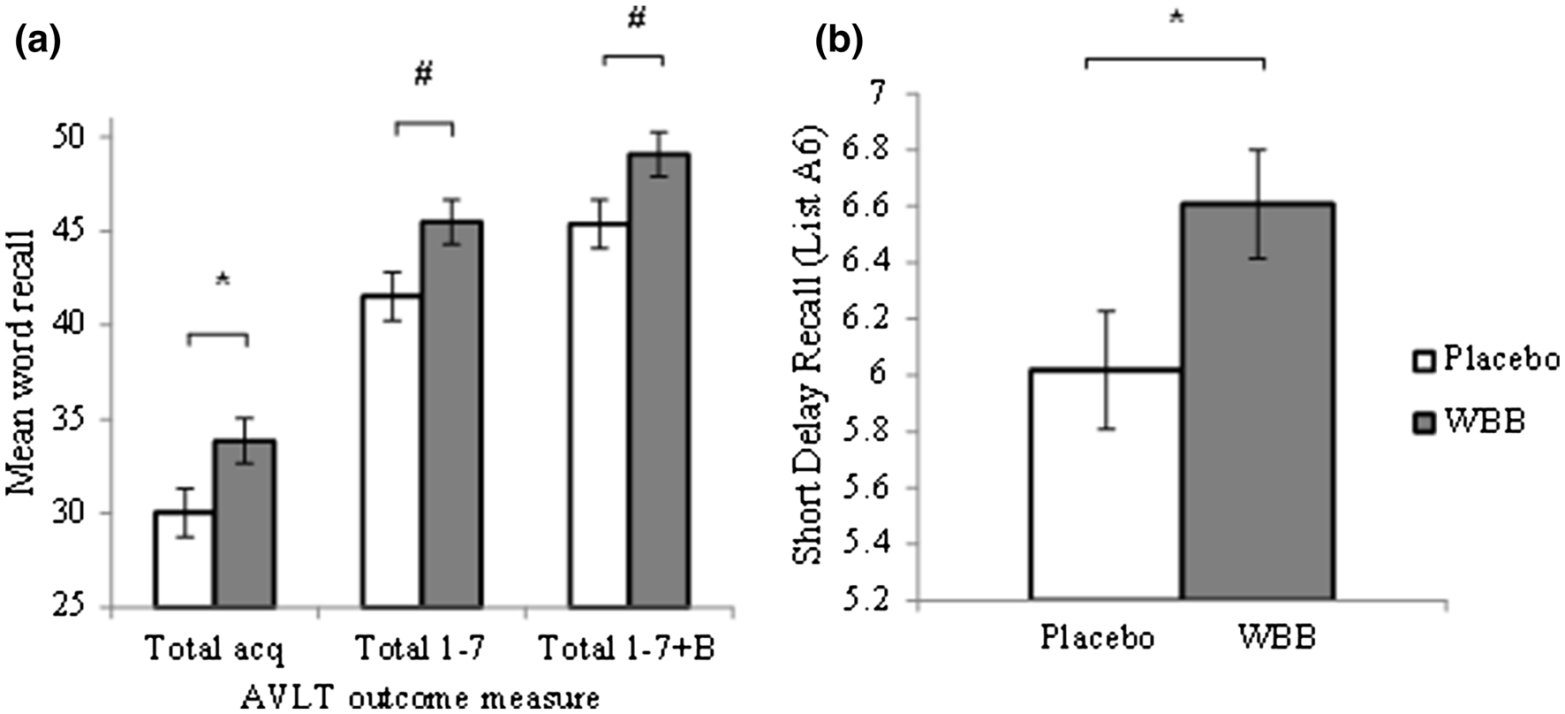
**a** WBB participants had significantly increased total acquisition performance compared to placebo participants following acute consumption. Total A1–A7 and Total A1–A7 + B word recall were also higher in WBB participants compared to placebo participants at the post-consumption time point. **b** Mean short delay word recall at the post-consumption time point was significantly higher for WBB participants compared to placebo. Asterisk denotes significance at the 5% level; Number sign denotes a trend between the 5 and 10% level

Drink was a significant predictor of post-consumption total acquisition performance (total recall A1–A5). Analysis revealed that WBB participants had higher total acquisition after their intervention than placebo participants (Table 2; Fig. 2a), suggesting a maintenance in performance for this group. A non-significant drink trend was also evident for total 1–7 (total recall A1–A7) and total 1–7 + B (total recall A1–A7 + B) performance such that WBB participants had better recall performance than placebo participants at the post-consumption time point (Table 2; Fig. 2a).

**Table 2:** Baseline and post-consumption performance for AVLT, TOWRE-2 and MANT outcome measures

Dependent variables	Baseline		Post-consumption (2 h)		LMM
	Placebo	WBB	Placebo	WBB	
	Mean (SD)	Mean (SD)	Mean (SD)	Mean (SD)	Drink fixed effect statistics
AVLT	(*n* = 24)	(*n* = 27)	(*n* = 24)	(*n* = 27)	
Word span	4.40 (1.44)	4.31 (1.38)	4.13 (1.23)	4.04 (1.17)	*F*(1,50) = 0.01, *p* = 0.94
Words learnt	5.25 (1.70)	4.86 (3.55)	3.79 (1.41)	4.21 (2.08)	*F*(1,51) = 1.52, *p* = 0.22
Final acquisition	9.44 (2.58)	9.19 (2.57)	7.92 (1.93)	8.25 (2.27)	*F*(1,51) = 1.01, *p* = 0.32
Total acquisition	36.92 (9.70)	35.11 (8.54)	30.75 (7.76)	33.25 (9.37)	*F*(1,52) = 4.69, *p* = 0.035*
Proactive interference	0.00 (1.73)	− 1.00 (1.96)	0.40 (1.58)	0.31 (1.20)	*F*(1,53) < 0.01, *p* = 0.99
Retroactive interference	2.52 (2.86)	1.36 (2.33)	1.76 (2.03)	1.59 (1.64)	*F*(1,53) < 0.01, *p* = 1.00
Total recall A1–A7	50.88 (13.68)	49.43 (12.51)	42.08 (11.14)	45.00 (10.92)	*F*(1,52) = 3.82, *p* = 0.056^#^
Total recall A1–A7 + B	55.28 (14.40)	54.43 (12.89)	45.79 (11.44)	48.71 (11.68)	*F*(1,52) = 3.22, *p* = 0.079^#^
Short delay recall	7.38 (1.94)	7.02 (1.71)	6.15 (1.55)	6.49 (1.48)	*F*(1,52) = 4.26, *p* = 0.04*
Long delay recall	7.04 (2.24)	6.82 (2.11)	5.25 (2.69)	5.93 (2.46)	*F*(1,52) = 1.99, *p* = 0.164
Delayed recall A6–A7	13.96 (4.74)	14.32 (4.68)	11.33 (4.26)	12.54 (4.33)	*F*(1,52) = 1.21, *p* = 0.28
Word recognition	11.04 (2.61)	10.56 (2.58)	9.20 (2.74)	9.21 (2.33)	*F*(1,51) = 0.48, *p* = 0.49
TOWRE-2					
SWE	60.67 (13.06)	64.74 (12.18)	61.42 (13.63)	65.70 (12.06)	*F*(1,51) = 0.113, *p* = 0.74
PDE	30.46 (12.37)	33.93 (9.52)	31.58 (12.98)	34.48 (9.99)	*F*(1,51) = 0.137, *p* = 0.71
MANT	(*n* = 25)	(*n* = 28)	(*n* = 25)	(*n* = 28)	
Accuracy (%)	74.89 (0.16)	75.52 (0.14)	82.63 (0.16)	81.53 (0.14)	*F*(1,49) = 0.17, *p* = 0.68
Reaction time (ms)	585.57 (102.31)	557.16 (132.84)	581.49 (71.66)	546.07 (127.72)	*F*(1,46) = 1.63, *p* = 0.21

LMM fixed effect statistics for Drink are shown. Significantly improved performance was observed on AVLT total acquisition, short delay recall and MANT reaction time

*Significant at < 0.05

^#^Trend < 0.1

Similarly, drink was found to be a significant predictor of performance on short delay trials (recall A6), such that WBB participants had significantly better short delay recall than placebo participants (Table 2; Fig. 2b). Again, this is indicative of maintenance of performance in the WBB group alongside task fatigue for the placebo group.

LMM analyses revealed that baseline performance significantly predicted post-consumption performance for 10 AVLT outcome measures [word span (*β* = 0.39), final acquisition (*β* = 0.49), total acquisition (*β* = 0.64), words learnt (*β* = 0.28), short delay (*β* = 0.60), long delay (*β* = 0.65), delayed recall (*β* = 0.58), recognition (*β* = 0.56), total recall A1–A7 (*β* = 0.62) and total recall A1–A7 + B (*β* = 0.64)].

#### TOWRE-2

There were no drink effects for sight reading efficiency (*F*(1, 51) = 0.11, *p* = 0.74) or phonemic decoding efficiency (*F*(1, 51) = 0.17, *p* = 0.71).

## Discussion

The current study administered a one-off dose of flavonoid-rich wild blueberry to typically developing 7–10-year-old schoolchildren following a randomised, placebo-controlled, parallel-groups design.

Beneficial effects of blueberry flavonoids were observed on an executive function task, the MANT, whereby WBB-treated participants exhibited quicker RT. No WBB effects were seen for accuracy. As expected across both treatment groups, a reduction in accuracy and RT was observed for the more cognitively demanding trials (incongruent, high load) of the MANT, when compared to less cognitively demanding trials (congruent, medium load, respectively). This reflects high internal validity and confirms that the task successfully manipulated cognitive demand, as participants performed worse on the more difficult trials.

Significantly quicker RTs were observed on 120 ms trials for WBB participants compared to placebo participants. Indeed, Simon and Berbaum [42] and Welford [43] previously noted that slower reaction times are to be expected in response to a slower stimulus duration on cognitive tasks due to a longer time required for information integration. However, findings suggest quicker RTs on trials of fast stimulus duration for WBB participants only, suggesting increased mental alertness on the faster 120 ms trials, without change to 500 ms trials (as supported by Fig. 1). It is also important to note that a similar effect was not observed in accuracy performance, suggesting the quickening of reaction times was without cost to accuracy. Interestingly, Whyte et al. [32] found that it was within the slower 500 ms trials of the MANT that WBB participants overcame the effects of the most cognitively demanding trials (incongruent, high load). Such an effect was not observed in the current study.

Beneficial WBB effects were also observed on the AVLT. As expected across both treatment groups, a reduction in verbal memory 2 h post-consumption was observed compared to baseline for a variety of facets of memory (word span, final acquisition, total acquisition, words learnt, short delay, long delay, delayed recall, recognition, total recall A1–A7 and total recall A1–A7 + B). This is indicative of an increase in fatigue and, therefore, forgetting, which is expected in a school environment, and has previously been observed by Whyte et al. [29]. In relation to this, the critical finding here was that consumption of WBB significantly attenuated forgetting for total acquisition (total recall A1–A5) and short delay recall (recall A6), as indicated by maintenance of post-consumption word recall following WBB, in comparison with the significant decline seen following placebo for these measures. As both drinks were matched for sugar content, this cannot be explained by a glucose effect. This beneficial effect for memory is consistent with Whyte et al. [29] who reported increased verbal memory performance in children at 1.15 h, 3 h and 6 h post-consumption following WBB consumption, using an identical 30 g WBB treatment and placebo as the current study, whilst children consuming placebo showed a decline in performance [29]. This further highlights the effect of WBB treatment in reducing fatigue, specifically in the domain of memory.

In addition to cognitive fatigue, between-session interference on the learning and recalling of information could also help to account for the general decline in memory performance. Words heard at the baseline session may have interfered with encoding and retrieval of new words at post-intervention. Interestingly, the present data provided no evidence of retroactive interference within session; new learning did not appear to alter retrieval of previously learned words in either treatment group. This effect was also observed by Whyte et al. [29] and implies a potentially different mechanism between stages of encoding and retrieval in children. Such interference parameters in a child population are yet to be determined and need further exploration. The general decline in memory performance could also be attributed to lunch consumption prior to testing. Research in adults indicates a post-lunch dip whereby lunch consumption negatively impacts upon cognitive performance postprandially [44], although the limited research conducted thus far in children demonstrates no effects of post-lunch dip on cognition, executive functions in particular [45, 46]. The absence of a pre-lunch test point means that a possible post-lunch dip effect remains speculative; this requires further investigation in children. It is also important to acknowledge that the cognitive benefits seen for WBB (relative to placebo) could be influenced by lunch, given that lunch was not standardised and did not occur at a standardised point prior to baseline testing (notwithstanding restriction of flavonoid intake). For practical reasons relating to the school environment the timing of the lunch was not fixed, and the content of lunch was not assessed, however, there is no reason to believe that there were significant differences in macronutrient intake between the WBB and placebo groups.

Despite the observed improvements to cognition following acute WBB intervention, no benefits were apparent for either sight word reading or phonemic decoding efficiency; however, it should be noted that children from this sample were good readers, as all performed well within or above the average range for their age group; therefore, these results cannot be generalizable to children with differing performance. Reading draws upon multiple aspects of cognition such as working memory, selective attention, and executive functions which must work together to achieve efficient reading. Therefore, it is plausible that a one-off dose of WBB may not be sufficient to elicit changes in reading, in comparison with the cognitive benefits seen here where aspects of cognition were assessed independently. Future research should consider the impact of sustained WBB supplementation on reading ability, such as a daily dose given chronically, to examine whether prolonged berry flavonoid supplementation is more beneficial to the complexity of cognitive domains that underpin reading ability. In addition, the task used to measure reading, the TOWRE-2, may not be sensitive enough to detect changes in reading ability attributable to WBB supplementation, or indeed potentially dietary interventions in general as to our knowledge it is the first time this task has been used in such a study. There is limited evidence for the effect of dietary interventions, namely polyunsaturated fatty acids, on word learning and memory using similar tasks; however, positive effects were only apparent in children with learning/behavioural difficulties or underperforming individuals [47, 48], reducing translation to typical readers. The TOWRE-2 measures the fluency and accuracy of orthography, or print-based words, within a short period of time (45 s). Essentially, this timed element measures participants’ automaticity of prior letter-sound correspondences and, in particular, may detract participants from making a correct response on the phonemic decoding subtest. To account for this potential lack of sensitivity, future research should focus on a select and more specific aspect of learning to read, such as word learning which focuses precisely on learning the mappings between orthography (a written word) and phonology (pronunciation). This is how children learn to read naturally so would not only examine the effect of WBB on a developmental aspect of reading requiring word memory and retrieval (similar to that measured by the AVLT although utilising a different modality), but will also allow generalisation of findings to the real world.

There is evidence to suggest that cognitive improvements are linked to increased cerebral blood flow [10]; however, definitive mechanisms are currently unknown, especially in a child population. Further work is needed to investigate the bioavailability of flavonoids within children’s bodies via non-invasive metabolic examinations, alongside cognitive assessment, to converge findings with a robust mechanism of action.

The current study demonstrated beneficial cognitive effects of acute WBB consumption on memory and attention in healthy 7–10-year-old children, although these benefits did not extend to reading ability. Such findings add to the growing body of evidence that flavonoids are beneficial for healthy brain function, and in this instance demonstrate the potential benefits of a 30 g freeze-dried WBB treatment, equivalent to a 240 g punnet or 1½ cups of fresh blueberries when relating findings to real-world applications, during critical developmental periods. Several animal and human studies have observed an increase in cognitive ability following acute blueberry interventions with adults [10, 11]; however, only four previous studies have explored the effects of berry fruits in children [26, 28, 29, 32]. Replications of such findings, alongside biological mechanisms of action, are desirable to further elicit the exact functioning of flavonoids within a child population, as well as considering the effects of chronic WBB consumption and more sensitive reading tasks when examining the effect of WBB on reading ability.
